# Evaluation of Formosa score and diagnostic sensitivity and specificity of four Asian risk scores for predicting intravenous immunoglobulin resistance in Kawasaki disease: a bivariate meta-analysis

**DOI:** 10.3389/fcvm.2023.1164530

**Published:** 2023-06-12

**Authors:** Wan-Ni Chiang, Po-Yu Huang, Ho-Chang Kuo, Ying-Hsien Huang, Ling-Sai Chang

**Affiliations:** ^1^ Division of Chinese Internal Medicine, Center for Traditional Chinese Medicine, Chang Gung Memorial Hospital, Taoyuan, Taiwan; ^2^ Department of Traditional Chinese Medicine, Kaohsiung Chang Gung Memorial Hospital and Chang Gung University College of Medicine, Kaohsiung, Taiwan; ^3^ Department of Pediatrics, Kaohsiung Chang Gung Memorial Hospital, Kaohsiung, Taiwan; ^4^ College of Medicine, Chang Gung University, Taoyuan, Taiwan

**Keywords:** diagnosis, Egami score, Formosa score, Kawasaki disease, Kobayashi score, meta-analysis, Sano score

## Abstract

**Background:**

In 2016, Lin et al. developed a prediction score of non-responsiveness to intravenous immunoglobulin (IVIG) in patients with Kawasaki disease (KD) (Lin et al., 2016). Various studies have attempted to validate the Formosa score, but inconsistent results have given us new opportunities and challenges. The aim of this meta-analysis is to explore the role of the Formosa score as a risk score in detecting IVIG-resistant KD patients and then compare the pooled sensitivity and specificity of four Asian risk scores, Egami, Formosa, Kobayashi, and Sano risk scores.

**Methods:**

A comprehensive search of Cochrane, Embase, and PubMed was conducted through 20 December 2021, using key terms relevant to the research question “What are the sensitivities and specificities of the four Asian predicting scores, Egami, Formosa, Kobayashi, and Sano, in Kawasaki disease patients with IVIG resistance?” The reference lists of the included studies were manually reviewed to identify pertinent references. A random-effects bivariate model was used to estimate the summary of sensitivity and specificity of the tools.

**Results:**

We found 41 relevant studies of the four Asian risk scores that were eligible to analyze for pooled accuracy. Eleven studies involving 5,169 KD patients reported the diagnostic performance of the Formosa score for the risk of IVIG resistance. The overall performance of the Formosa score was as follows: pooled sensitivity, 0.60 [95% confidence interval (CI), 0.48–0.70]; pooled specificity, 0.59 (95% CI, 0.50–0.68); and area under the hierarchical summary receiver operating characteristic curve, 0.62. The Formosa score exhibited the highest sensitivity 0.76 (95% CI, 0.70–0.82) for detecting IVIG-resistant KD patients among the 21,389 children included in the 41 studies. In terms of specificity estimates, Formosa had the lowest specificity of 0.46 (95% CI, 0.41–0.51).

**Conclusion:**

Patients at high risk for IVIG resistance may receive adjunctive treatment to reduce coronary lesions and thus also cardiovascular morbidity. Among all of the included studies, we found Formosa score to have the best sensitivity (0.76) but unsatisfactory specificity (0.46) for predicting IVIG resistance in Kawasaki disease. In the future, network meta-analysis should also incorporate the accuracy of the new scores after they have undergone a certain degree of validation around the world.

**Systematic Review Registration:**

https://www.crd.york.ac.uk/PROSPERO/, PROSPERO CRD42022341410.

## 1. Introduction

The prevalence of Kawasaki disease (KD) is highest in Asia (1). National surveys in Japan, South Korea, and Taiwan have all confirmed this finding. KD is not just acute vascular inflammation, as long-term follow-up has found that it affects immunity and the development of allergic diseases (2–5). Intravenous immunoglobulin (IVIG) resistance is a term that was developed after the invention of IVIG treatment for KD (6). Approximately 10%–20% of patients still experience persistent or recurrent fever after completing the initial IVIG administration and are thus classified as unresponsive to IVIG treatment. This group of patients is at an increased risk of developing coronary artery lesions (CAL) (7, 8). Many scoring systems have been used to predict the risk of IVIG resistance (9). In particular, the scoring systems in Asia have been repeatedly verified for a long time in the hopes of providing initial treatment guidelines for high-risk patients in Asia (10, 11).

Many scoring systems have been developed to predict IVIG resistance in KD, such as Egami, Formosa, Kobayashi, and Sano scores (12). However, their efficacy needs to be validated given the regional and racial population (12). Fabi et al. enrolled both Caucasian and Asian children and determined that Formosa score had the highest predictive efficacy for CAL risk (13). The Kobayashi score had a sensitivity of 64.0% and specificity of 62.5% in a total of 257 patients. However, when applied to seven Asian patients, the Kobayashi score had a sensitivity of 100% and a specificity of 75% (13).

IVIG resistance scoring systems can help clinicians identify high-risk KD patients who may benefit from so-called “rescue therapies,” such as IVIG plus prednisolone or IVIG plus cyclosporine (10, 11). Adopting additional treatment before the initial use of IVIG could potentially reduce the incidence of CAL in IVIG-resistant KD patients (14).

According to the Kobayashi score, cutoff points and score points for each variable are as follows: sodium ≦133 mmol/L, 2 points; days of illness at initial treatment ≤4, 2 points; aspartate aminotransferase (AST) ≥100 IU/L, 2 points; % neutrophils ≥80%, 2 points; C-reactive protein (CRP) ≥10 mg/dL, 1 point; age ≤12 months, 1 point; and platelet count ≤30.0×10^4^/mm^3^, 1 point. Patients with a total of 4 or more points are identified as being at high risk for IVIG resistance. This score has a sensitivity of 86% and specificity of 68% in predicting IVIG resistance (7). According to the Egami score, based on the odds ratios of significant predictors, 1 point is assigned for infants younger than 6 months, before 4 days of illness, platelet count of ≤30 × 10^10^/L, and CRP of ≥8 mg/dl, respectively. Two points are assigned for alanine transaminase (ALT) 80 IU/L. Using a cutoff point of 3 or more points with this prediction score, it could identify the IVIG-resistant group with a sensitivity of 78% and specificity of 76% (15). According to the Sano score, the criteria for at least two of the three predictors (CRP ≥7 mg/dl, total bilirubin ≥0.9 mg/dl, or AST ≥200 IU/L) are considered to be clinically useful for detecting non-responsiveness to IVIG in patients with acute KD before treatment, with a sensitivity of 77% and specificity of 86% (16). According to the Formosa score, cutoff points and score points for each variable are as follows: albumin <3.5 g/dl, 1 point; neutrophil percentage ≥60%, 2 points; and positive lymphadenopathy, 1 point. Patients with scores of ≥3 points are identified as being at high risk for IVIG resistance. Their sensitivity and specificity have been shown to be 90.9% and 81.3%, respectively (17).

In this study, we compared the predictive efficacy of the Egami, Formosa, Kobayashi, and Sano scoring systems using a bivariate meta-analysis.

## 2. Methods

We conducted this study in accordance with the guidelines of the preferred reporting items for systematic reviews and meta-analysis of diagnostic test accuracy studies (PRISMA-DTA) (18). We formulated the following patient, index test, comparison, outcome (PICO) question: “What are the sensitivities and specificities of four Asian predicting scores, Egami, Formosa, Kobayashi, and Sano, in Kawasaki disease patients with intravenous immunoglobulin resistance?” The definition of IVIG resistance varied according to different studies (Table 1) (19). We registered the study protocol at the International Prospective Register of Systematic Reviews (PROSPERO CRD42022341410).

**Table 1A:** Clinical characteristics of the included studies: included studies with the Formosa score.

	Design	Study author	Year	Country	Scores	KD patients	IVIG-resistant rate (%)	Criteria of IVIG resistance	Diagnostic criteria of KD	IVIG	Exclusion
1	Retrospective	Lin	2016	Taiwan	Formosa	238	12.2	Persistent fever beyond 24 h after IVIG or recrudescent fever	Japanese	2 g/kg for 1 day or 1 g/kg for 2 days	10 IVIG-responsive KD patients did not receive a test for albumin levels
2	Retrospective	Song	2017	China	Egami Formosa Kobayashi	1,163	9	(1) Fever persisting for 48 h after IVIG (temperature >38°C) and (2) recrudescent fever within 7 days of IVIG	Japanese	2 g/kg/day	(1) Recurrent cases, (2) receipt of initial treatment before hospitalization, (3) presence of other vascular inflammatory diseases, and (4) incomplete clinical data
3	Prospective	Qian	2018	China	Egami Formosa Kobayashi Sano	504	5	Fever at >36 h after completion of the initial IVIG	AHA	2 g/kg/day	No IVIG
4	Retrospective Prospective	Arslanoglu	2019	Turkey	Egami Formosa Kobayashi	100	15	Persistent or recrudescent fever for at least 36 h after completion of the first IVIG	AHA	2 g/kg, infusion in 12 h	
5	Prospective	Shao	2019	China	Egami Formosa Kobayashi Sano	393	13.7	Fever over 36 h after the end of the IVIG infusion or recurrent fever	AHA	2 g/kg of IVIG for 24 h	Initial IVIG treatment at other medical facilities or did not receive IVIG treatment between 4 and 10 days from fever onset; IVIG treatment had been initiated before blood sampling; incomplete laboratory data or lack of follow-up results
6	Multicenter retrospective	Fabi	2019	Italy	Egami Formosa Kobayashi	257	16.7	Persistent/recrudescent fever for at least 36 h but for no longer than 7 days after the completion of the first IVIG	AHA	2 g/kg in a single infusion	Incomplete data, late treatment, and not treated
7	Retrospective	Wang	2020	China	Egami Formosa Kobayashi	644	19.3	Recrudescent or persistent fever for ≧36 h after the end of the IVIG	AHA	2 g/kg in 1 day or 1 g/kg separated between 2 days	A severe lack of laboratory results, and patients were never treated with IVIG during their hospitalization. Disagreement with the AHA guidelines
8	Retrospective	Oztarhan	2020	Turkey	Egami Formosa Kobayashi Sano	259	12.4	Recurrent or persistent fever for at least 36 h after the end of IVIG	AHA	2 g/kg as a single infusion	Missing file data or with a diagnosis other than KD
9	Retrospective	Huang	2021	Taiwan	Egami Formosa Kobayashi	84	11.0	After completion of the first course of IVIG, patients had persistent fever for >24 h or developed recrudescent fever within 7 days	AHA	2 g/kg for 1 day or 1 g/kg for 2 days	
10	Retrospective	Ummusen	2021	Turkey	Egami Formosa Kobayashi Sano	129	12.4	Persistent or recurring fever for at least 36 h after the end of the IVIG infusion	AHA		
11	Retrospective	Liu	2021	China	Egami Formosa Kobayashi Sano	1,398	11.3	A persistent or recurrence of fever of ≥38°C at any time from 36 h to 2 weeks after initial IVIG	Japanese	2 g/kg	(1) Incomplete KD and other confounding diseases, such as toddler's idiopathic arthritis, (2) rehospitalized due to recurrence of KD, (3) diagnosed with KD outside the hospital and receiving IVIG treatment, and (4) incomplete clinical data

AHA, American Heart Association; h, hours; CRP, C-reactive protein; IVIG, intravenous immunoglobulin; KD, Kawasaki disease.

**Table 1B:** Clinical characteristics of the included studies: included studies without the Formosa score.

Design	Study author	Year	Country	Scores	KD patients	IVIG-resistant rate (%)	Criteria of IVIG resistance	Diagnostic criteria of KD	IVIG	Exclusion
Retrospective	Egami	2006	Japan	Egami	320	13	A responder had resolution of fever (<37.5°C) and a fall in CRP by more than 50% within 48 h after initial IVIG	Japanese	Single 2 g/kg/dose	Cardiovascular complications at initial treatment
Retrospective	Kobayashi	2006	Japan	Kobayashi	676	22.0	Fever persisting beyond 24 h or recrudescent fever after an afebrile period	Japanese	1 g/kg per day over 12 h for 2 consecutive days	Other infectious disease known to mimic KD or atypical KD. Cardiovascular complications before initial treatment; 2 patients did not complete IVIG treatment because of hypotension
Retrospective	Sano	2007	Japan	Sano	112	20	Persistent fever (≥37.5°C over 24 h) after finishing IVIG	Japanese	1 g/kg/day of IVIG was administered for 2 days	
Retrospective	Tremoulet	2008	America	Egami	362	9.8–20	Persistent or recrudescent fever (*T* ≥ 100.4° F rectally or orally) for at least 48 h but not longer than 7 days after completion of the first IVIG	AHA	2 g/kg	
Retrospective	Seki	2011	Japan	Kobayashi (cutoff of ≧5 points)	1,626	22.8	Given additional rescue therapy because of persistent fever lasting for more than 24 h after the end of the IVIG infusion, or recrudescent fever despite an afebrile period after treatment	Japanese	1 g/kg/day for 2 days or 2 g/kg/day for 1 day	Cardiovascular complications before the initial treatment or who received steroids as part of the initial therapy
Retrospective	Sleeper	2011	North America	Egami Kobayashi Sano	78 62 56	14	Fever of at least 38.3°C without another likely source at >36 h after completion of the initial IVIG	AHA	2 g/kg	Withdrawal
Retrospective	Park	2013	Korea	Egami Kobayashi Sano	309	9.7	Fever continued for over 36 h or who had recrudescent fever (temperature ≥38.0°C axillary or rectally)	AHA	2 g/kg	
Retrospective	Fu	2013	China	Egami Kobayashi	1,177	17.9	Persistent or recurrent fever at any time from 48 h to 2 weeks after initial IVIG treatment and with at least one of the standard diagnostic criteria	Japanese	1 g/kg, twice, 2 g/kg, once, 400–500 mg/kg, 3–5 days	(1) Clinical or laboratory evidence was not complete, (2) children were diagnosed after the first 10 days, (3) chronic KD and visited our hospital for coronary artery lesion, or (4) children were diagnosed with KD in other hospitals and had been treated with IVIG
Retrospective	Davies	2015	United Kingdom	Kobayashi	59	32.2	Match the definition of Kobayashi			Clinical or laboratory evidence was not complete
Retrospective	Kim	2016	Korea	Egami Kobayashi Sano	703	16.8	Received more than one dose of IVIG due to persistent or recrudescent fever		A single dose 2 g/kg	The data were insufficient, or had no fever, and therefore, had not received IVIG treatment
Retrospective	Tang	2016	China	Egami Kobayashi	910	5	Persistent or recrudescent fever for ≥36 h after the initial IVIG	AHA	2 g/kg	IVIG after the tenth day of illness and presence of another disease known to mimic KD
Retrospective	Sanchez	2016	Spain	Egami	305	16.4	Required a second dose of IVIG	AHA		Missing data, those whose fever duration data were missing, those who, were admitted for a second opinion, and those whose informed consent was missing or incomplete.
Retrospective	Kanamitsu	2016	Japan	Egami Kobayashi Sano	183 183 163	20.8	The necessity for additional IVIG or immunosuppressive medications	Japanese	A single dose 2 g/kg	Missing laboratory data
Retrospective	Shin	2017	Korea	Egami Kobayashi	204	50	Required a second dosage of IVIG or steroid therapy because of a persistent or reappearance of fever within 36 h after the initial IVIG treatment		2 g/kg	Younger than 2 years old
Retrospective	Takeshita	2017	Japan	Egami Kobayashi Sano	437	21.3	Persistent fever lasting for >24 h after the completion of IVIG or recrudescent fever	Japanese	2 g/kg/day	Incomplete KD or any other disease or with CAL before the treatment
Retrospective	Berdej-Szczot	2017	Poland	Kobayashi	73	11	The persistence or recurrence of fever for >36 h after IVIG administration	AHA	Cumulative dose of 2 g/kg	
Retrospective	Chbeir	2018	France	Egami Kobayashi (cutoff of ≧5 points) Sano	149 152 123	28.7	The persistence of fever (temperature >38.0°C) for 48 h after the IVIG	AHA	2 g/kg	A diagnosis other than KD was established during follow up
Retrospective	Arane	2018	Israel	Egami Kobayashi Sano	236 219 223	18.4	A persistent fever after the first dose of IVIG, which was defined as having fever after 24–72 h	AHA	2 g/kg	Admitted during the sub-acute phase
Retrospective	Gámez-González	2018	Japan	Kobayashi Sano	419	24.1	Fever was not resolved (defined as having an axillary temperature above 37.5°C) within 48 h of initial IVIG therapy start	Japanese	2 g/kg for 24 h	
Retrospective	Jakob	2018	Germany	Egami Kobayashi Sano	301	15.6	Fever persisting for longer than 36 h and therefore given a second dose of IVIG	AHA		Failure to comply with the clinical case definition; IVIG untreated patients; steroid administration concurrent with the first IVIG therapy; and where neither “yes” nor “no” was ticked on the questionnaire, uncertain steroid exposure.
Retrospective	Fernandez-Cooke	2019	Spain	Egami Kobayashi (cutoff of ≧5 points) Sano	606	15.7	Persistence of fever for 36 h after the end of IVIG infusion	AHA		Older than 16 years at the time of diagnosis, those patients found to be duplicated in the database and patients with a final alternative diagnosis no IVIG
Retrospective	Grignani	2019	Singapore	Egami Kobayashi Sano	122	12	Recrudescent or persistent fever of more than 38°C more than 48 h after completion of IVIG	AHA	2 g/kg over 1 day	Incomplete data
Retrospective	Tan	2019	China	Egami Kobayashi Sano	5,277	6.6	A persistence or recurrence of fever of >37.3°C at any time during 48 h to 2 weeks after initial IVIG	Japanese		Incomplete KD and other diseases had been given IVIG treatment in other medical institutions before admission and who didn’t receive IVIG treatment
Retrospective	Ha	2020	Korea	Egami Kobayashi Sano	555	34.8	Febrile for >48 h after receiving IVIG	AHA	A single 2 g/kg dose	
Retrospective	Shashaani	2020	Iran	Egami Kobayashi Sano	363	24	A persistent fever after the first IVIG, which was defined as having a fever after 24–72 h	AHA	2 g/kg	Admitted during the sub-acute phase
Retrospective or prospective	Piram	2020	France	Egami Kobayashi Sano	320 334 211	11.9 14.7 2.8	The need for a second course of IVIG or second-line treatment (after the first IVIG infusion) with corticosteroids or anti-tumor necrosis factor agent	AHA		Unclassified and doubtful KD adult patients
Cross-sectional retrospective	Edraki	2020	Iran	Egami Kobayashi Sano	121 113 121	13.2	Needed more than 2 g/kg IVIG to stop fever, recurrent fever, and any patient who needed steroid or infliximab	AHA	2 g/kg	Did not have a thorough follow-up
Prospective observational study	Ishikawa	2021	Japan	Egami Kobayashi Sano	31	32.3	Persistent or recrudescent fever of ≥38.0°C for at least 36 h after the end of IVIG	AHA	2 g/kg	
Retrospective	Faim	2021	Portugal	Egami Kobayashi Sano	39 34 25	21	Fever persisted for 36 h after IVIG administration	AHA		Transferred from outlying centers with a diagnosis of KD and managed at these institutions did not have the necessary data
Retrospective	Jarutach	2021	Thailand	Egami Kobayashi Sano	130	13	A recrudescent or persistent fever for at least 36 h after the end of the first IVIG	AHA	2 g/kg of IVIG infusion over 10–12 h	IVIG at doses of less than 2 g/kg

We first performed a systematic literature search in all fields in international electronic databases, including Cochrane, Embase, and PubMed (20). We applied the combinations of keywords used, respectively, with the “Kobayashi score,” “Egami score,” “Sano score,” “Formosa score,” “sensitivity,” and “specificity” to identify relevant articles (21). Our search only included papers published in the English language. The reference lists of the included studies were manually reviewed to identify cited articles of these four Asian scores (7, 15–17). Original articles would be included in this meta-analysis if they met the following criteria: (1) examination of patients with KD; (2) assessment of the sensitivity and specificity of the Egami, Formosa, Kobayashi, or Sano scores; and (3) received treatment with a total IVIG of 2 g/kg including one single dose or 1 g/kg per day for 2 consecutive days. When the study reported Kobayashi score with a cutoff value of ≧4 and ≧5, we recorded the value with the cutoff of ≧4 for analysis. We ruled out case reports and studies that predicted IVIG resistance with a predictive score after diagnosing KD and then prescribing different treatments. Studies that did not report sensitivity or specificity values and sample sizes were excluded. Two investigators (Wan-Ni Chiang and Dr. Ling-Sai Chang) independently extracted data from each included study by using a predesigned data extraction form, including the authors, publication year, the country where the study was conducted, study design, age, percentage of male participants, number of participants, and cutoff value for the analysis of sensitivity and specificity. The same two investigators (Wan-Ni Chiang and Dr. Ling-Sai Chang) independently performed a systematic literature search and evaluated all relevant studies for eligibility criteria. Any disagreement was resolved through discussion.

After the full systematic literature search was performed, we used bivariate statistical analysis to obtain the logit-transformed sensitivity and specificity of the Formosa score. To estimate the summary of sensitivity and specificity, we adopted a random-effects bivariate model. All analyses were performed using Stata version 17.0 (StataCorp LP, College Station, TX, United States) with meqrlogit for network calculation based on the ANOVA model proposed by Nyaga et al.; metandi for making the graph of the hierarchical summary receiver operating characteristic curve (HSROC); midas for calculating sensitivity and specificity of the Formosa score, heterogeneity measures, *I*^2^ estimation, the area under the curve (AUC), and subgroup calculation; and melogit for comparing the Formosa score and the other three Asian scores’ user-written commands (20, 22). Furthermore, we accessed the publication bias for evaluating the accuracy of the Formosa score using Deeks’ funnel plot asymmetry test (23).

We adopted the revised Quality Assessment of Diagnostic Accuracy Studies to evaluate the methodological quality of selected studies according to four domains comprising 14 items rated as “yes,” “no,” or “unclear” (24).

## 3. Results

### 3.1. Study selection

**Figure 1:**
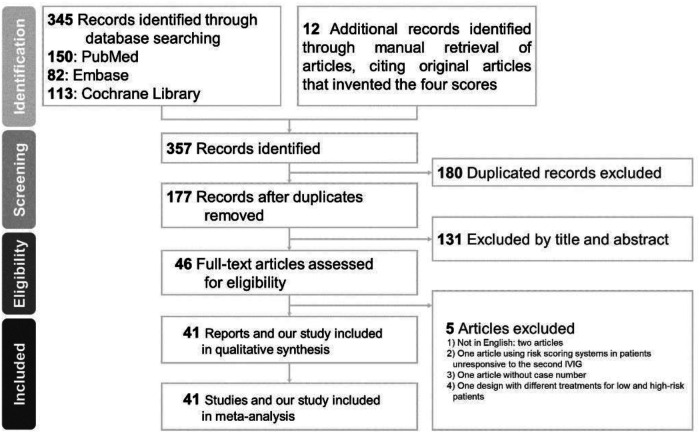
Flow diagram of literature retrieval for reporting the study selection process.

We identified a total of 345 articles through database searching (PubMed = 150, Embase = 82, Cochrane Library = 113) and 12 additional records through manual retrieval of articles, citing the original articles that invented the four scores (8, 16, 25–34). Of the 177 records initially identified through title and abstract screening after removing duplicates, 131 were removed for failing to fulfill the inclusion criteria (Figure 1). Further full-text assessment of the potential 46 articles led to the exclusion of five studies, which were excluded for the following reasons: two not in English, one using risk scoring systems in patients unresponsive to the second IVIG, one without case number, and one design with different treatments for low- and high-risk patients (35–39). Ultimately, a total of 41 studies were included in the network meta-analysis.

### 3.2. Study characteristics

A total of 41 articles met the inclusion criteria in Table 1, which provides broad details of the studies. Eleven studies were included in both the bivariate meta-analysis for the Formosa score (Table 1A) and network meta-analysis for the four Asian scores. All included studies were written in English. The median number of patients was 305 (interquartile range, IQR: 125.5–580.5), while the median prevalence of IVIG resistance was 15.7% (IQR: 12.1%–21.2%). Thirty-four studies with 18,170 KD patients evaluated the Egami score; 11 studies with 5,169 KD patients evaluated the Formosa score; 36 studies with 20,006 KD patients evaluated the Kobayashi score; and 25 studies with 12,970 patients evaluated the Sano score.

Of the 41 studies, three conducted prospective studies, two conducted retrospective or prospective studies, and the remaining 36 were retrospective studies. All studies provided detailed information on the reference standard for diagnosing IVIG resistance. The definition of reference was persistent or recrudescent fever at least 24, 36, or 48 h after completion of the first IVIG or the necessity for additional IVIG or immunosuppressive medications. These 41 studies were conducted between 2006 and 2021. Four of the studies excluded incomplete KD patients (7, 31, 40, 41). Furthermore, four studies excluded cardiovascular complications before or at initial treatment (7, 15, 41, 42). While three studies adopted thresholds of ≧5, other studies evaluating Kobayashi score used Kobayashi-specified thresholds (≧4) to classify the results (28, 29, 42). This study consists of four different Asian scores, namely, 3 studied Egami score, 1Formosa, 4 Kobayashi, 1 Sano, 3 Egami + Kobayashi, 1 Kobayashi + Sano, 5 Egami + Formosa + Kobayashi, 18 Egami + Obayashii + Sano, and 5 Egami + Formosa + Kobayashi + Sano scores.

### 3.3. Results of meta-analysis for the sensitivity and specificity of the Formosa score

**Figure 2:**
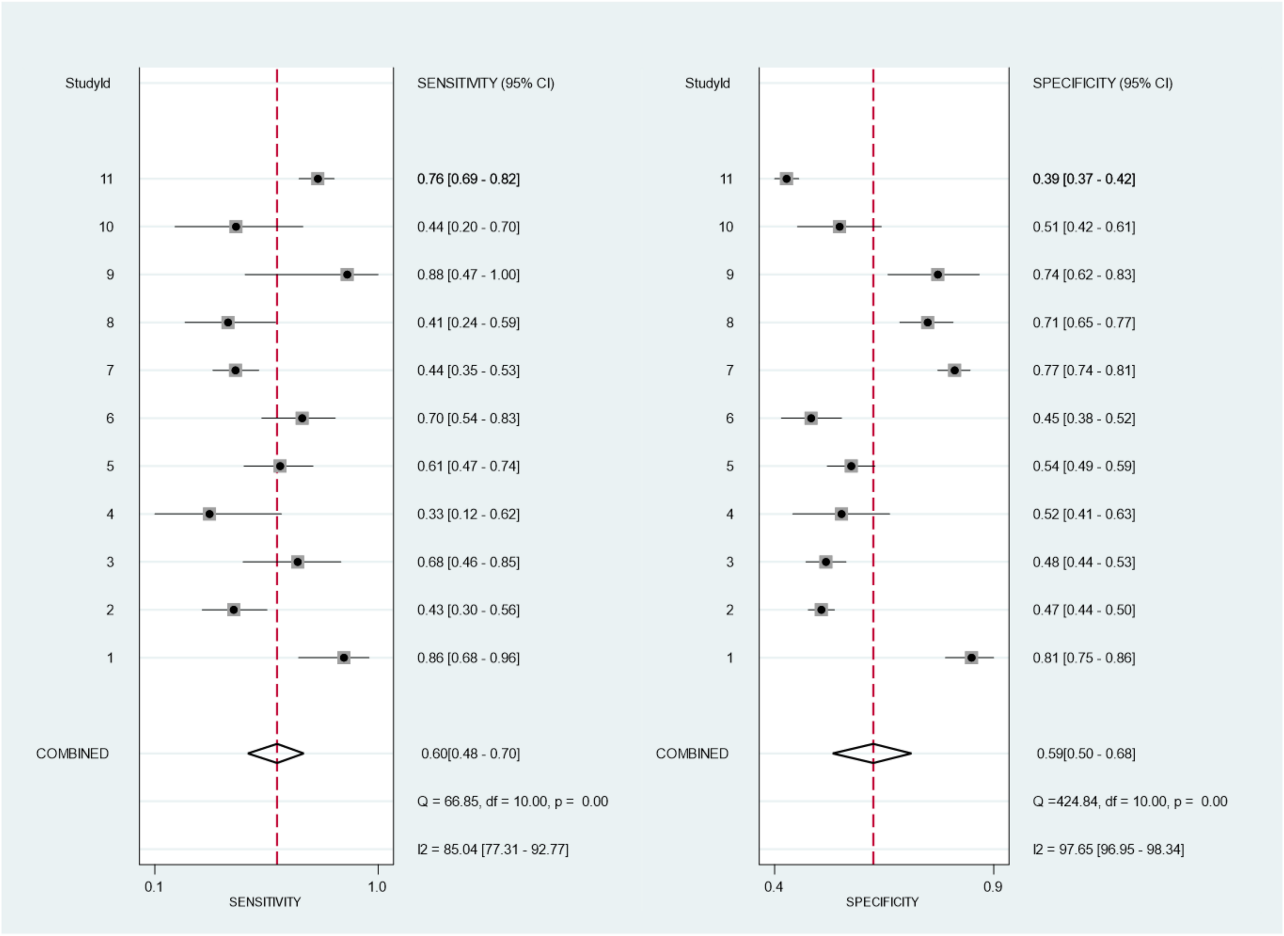
Bivariate meta-analysis of the Formosa score for pooled sensitivity and specificity of 11 included studies. The study ID is identified in Table 1A.

**Figure 3:**
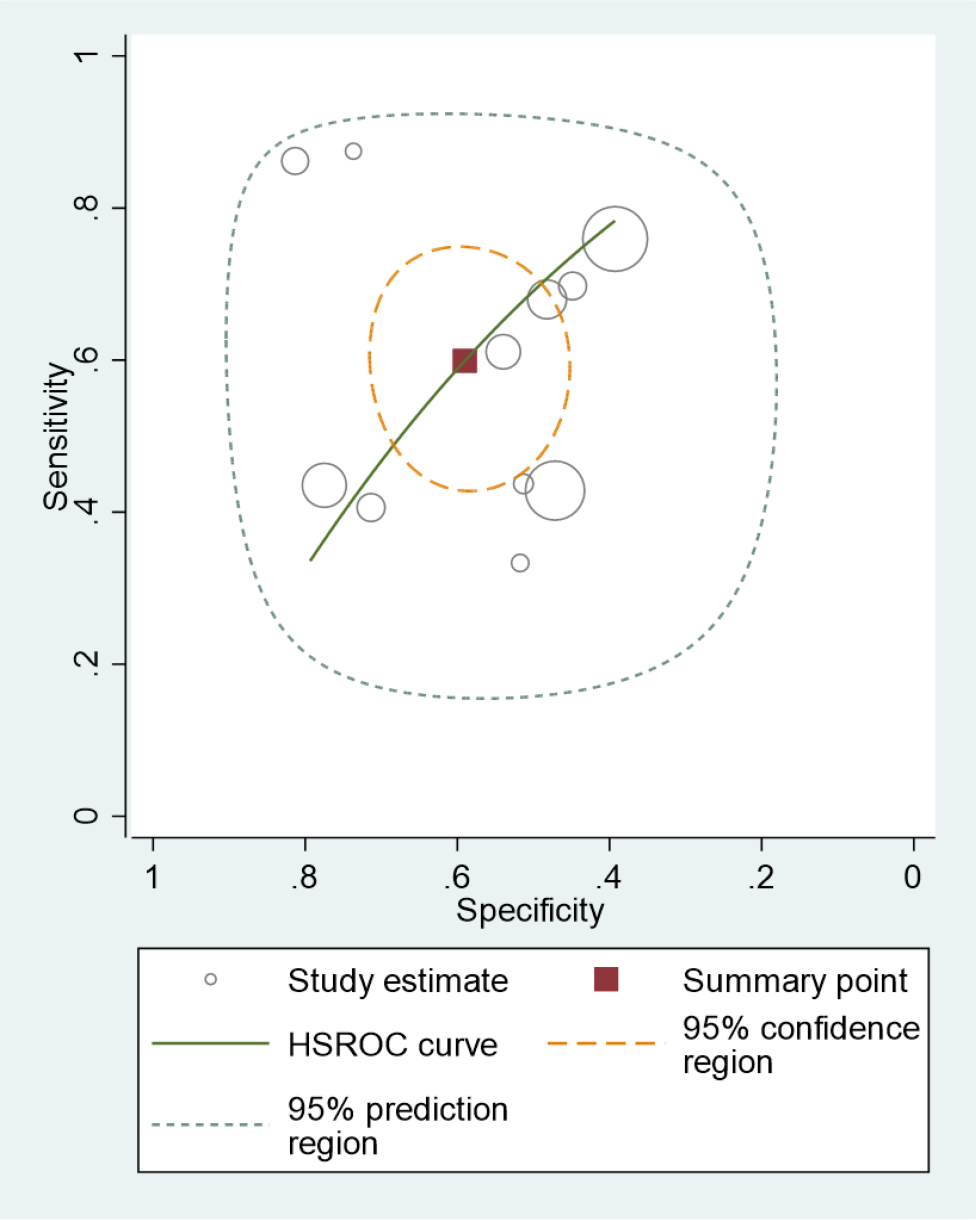
Hierarchical summary receiver operating curve (HSROC) of the sensitivity vs. the specificity of the performance of the Formosa score for predicting intravenous immunoglobulin resistance in Kawasaki disease patients. Each included study is represented by a circle; squares represent the summary test accuracy.

In the analysis, we identified 11 studies involving 5,169 KD patients that reported the diagnostic performance of the Formosa score for IVIG-resistant risk (12, 13, 17, 30, 34, 40, 43–47). Figure 2 shows the overall performance of Formosa score: pooled sensitivity, 0.60 [95% confidence interval (CI), 0.48–0.70]; pooled specificity, 0.59 (95% CI, 0.50–0.68); and area under the summary receiver operating characteristic curve (SROC), 0.62, as illustrated in Figure 3.

The potential sources of significantly statistical heterogeneity were IVIG-resistant rate, the definition of IVIG resistance, and the diagnostic criteria of KD. The factors that may explain the heterogeneity must be further evaluated by subgroup analysis (Table 2). The meta-regression suggested that the sensitivity and specificity of Asian studies (*n* = 7) were not significantly greater than that of the non-Asian studies (*n* = 4) (sensitivity in Asian, 0.65, with 95% CI, 0.53–0.78, and sensitivity in non-Asian, 0.48, with 95% CI, 0.30–0.67, *p* = 0.16; specificity in Asian, 0.61, with 95% CI, 0.50–0.72, and specificity in non-Asian, 0.55, with 95% CI, 0.40–0.71, *p* = 0.35). A trend of lower sensitivity in Turkey was also found (sensitivity in Turkey, 0.39, with 95% CI, 0.53–0.78, and sensitivity in non-Turkey, 0.66, with 95% CI, 0.30–0.67, *p* = 0.11). No significant difference was observed between subgroups according to ethics, diagnostic criteria, total scores, or IVIG-resistant rate, as shown in Table 2 (*p* > 0.05).

**Table 2:** Meta-regression results for the diagnostic performance of the Formosa score for predicting IVIG resistance.

	Non-Asian	95% CI	Asian	95% CI	*p*-value
Number of studies	4		7		
Sensitivity	0.48	0.30–0.67	0.65	0.53–0.78	0.16
Specificity	0.55	0.40–0.71	0.61	0.50–0.72	0.35
	Non-Turkey	95% CI	Turkey	95% CI	*p*-value
Number of studies	8		3		
Sensitivity	0.66	0.55–0.76	0.39	0.20–0.58	0.11
Specificity	0.59	0.49–0.70	0.59	0.41–0.76	0.65
	Non-China Han	95% CI	China Han	95% CI	*p*-value
Number of studies	4		7		
Sensitivity	0.48	0.30–0.67	0.65	0.53–0.78	0.44
Specificity	0.55	0.40–0.71	0.61	0.50–0.72	0.93
	IVIG-resistant rate ≦medium 12.4	95% CI	IVIG-resistant rate >medium 12.4	95% CI	*p*-value
Number of studies	7		4		
Sensitivity	0.64	0.51–0.77	0.53	0.35–0.71	0.75
Specificity	0.60	0.48–0.71	0.58	0.43–0.73	0.67
	Studies involving four scores	95% CI	Studies not involving four scores	95% CI	*p*-value
Number of studies	5		6		
Sensitivity	0.60	0.44–0.76	0.60	0.44–0.75	0.66
Specificity	0.53	0.40–0.66	0.64	0.53–0.75	0.80
	KD diagnosis by Japanese criteria	95% CI	KD diagnosis by AHA	95% CI	*p*-value
Number of studies	3		8		
Sensitivity	0.70	0.53–0.86	0.55	0.42–0.68	0.69
Specificity	0.57	0.40–0.74	0.60	0.49–0.70	0.52

AHA, American Heart Association; CI, confidence interval.

### 3.4. Results of network meta-analysis of the four Asian scores

**Figure 4:**
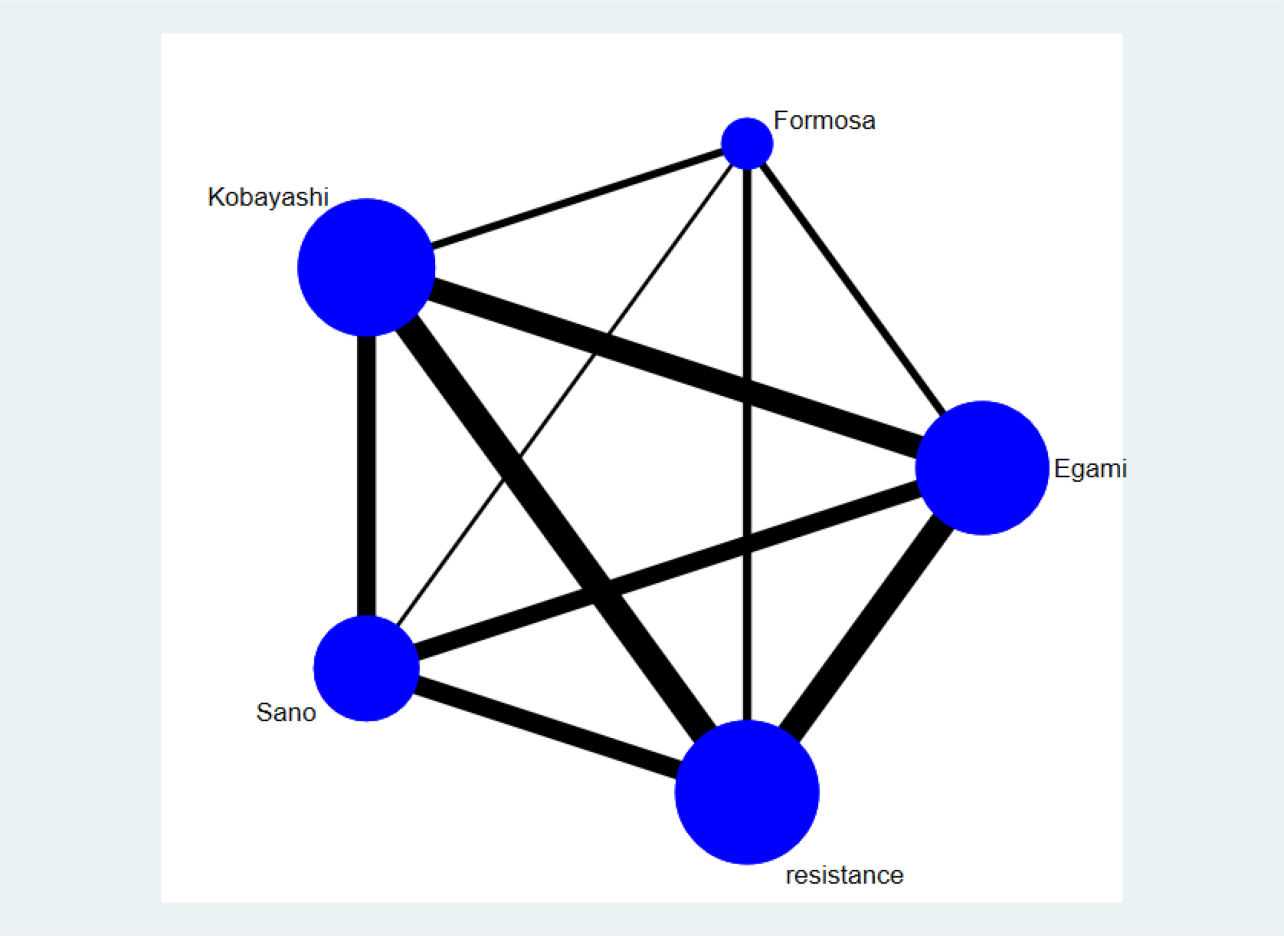
The network graph of the four scores only represents the comparisons of included studies. The size of the node represents the number of studies; the thickness of the line showing direct comparisons represents the number of studies. Resistance by the definition of each study is used as a reference standard.

The network graph of the relationship between the four Asian scores and reference standard is shown in Figure 4. Of the children included in the network meta-analysis, 21,389 with a confirmed diagnosis of KD by the American Heart Association (AHA) or Japanese criteria were included in the comparison of sensitivity and specificity among the four Asian scores (15, 19). The current study enrolled 41 studies, and the results of the four scoring systems in predicting IVIG resistance are shown in Table 3. Based on the 41 studies, we suggest that the Formosa score has the highest sensitivity in predicting IVIG resistance among the four scores. The Formosa score has the lowest specificity. In contrast, Egami and Kobayashi score had high specificities.

**Table 3:** Results of network meta-analysis of four Asian scores.

By meqrlogit command	Four Asian scores		95% confidence interval		*p*-value compared to Formosa score by melogit command
Sensitivity	Egami	0.39	0.32	0.46	<0.01*
	Formosa	0.76	0.70	0.82	
	Kobayashi	0.46	0.39	0.53	<0.01*
	Sano	0.36	0.30	0.43	<0.01*
Specificity	Egami	0.83	0.80	0.86	<0.01*
	Formosa	0.46	0.41	0.51	
	Kobayashi	0.81	0.78	0.84	<0.01*
	Sano	0.71	0.67	0.75	<0.01*

* *p* < 0.05.

### 3.5. Publication bias

**Figure 5:**
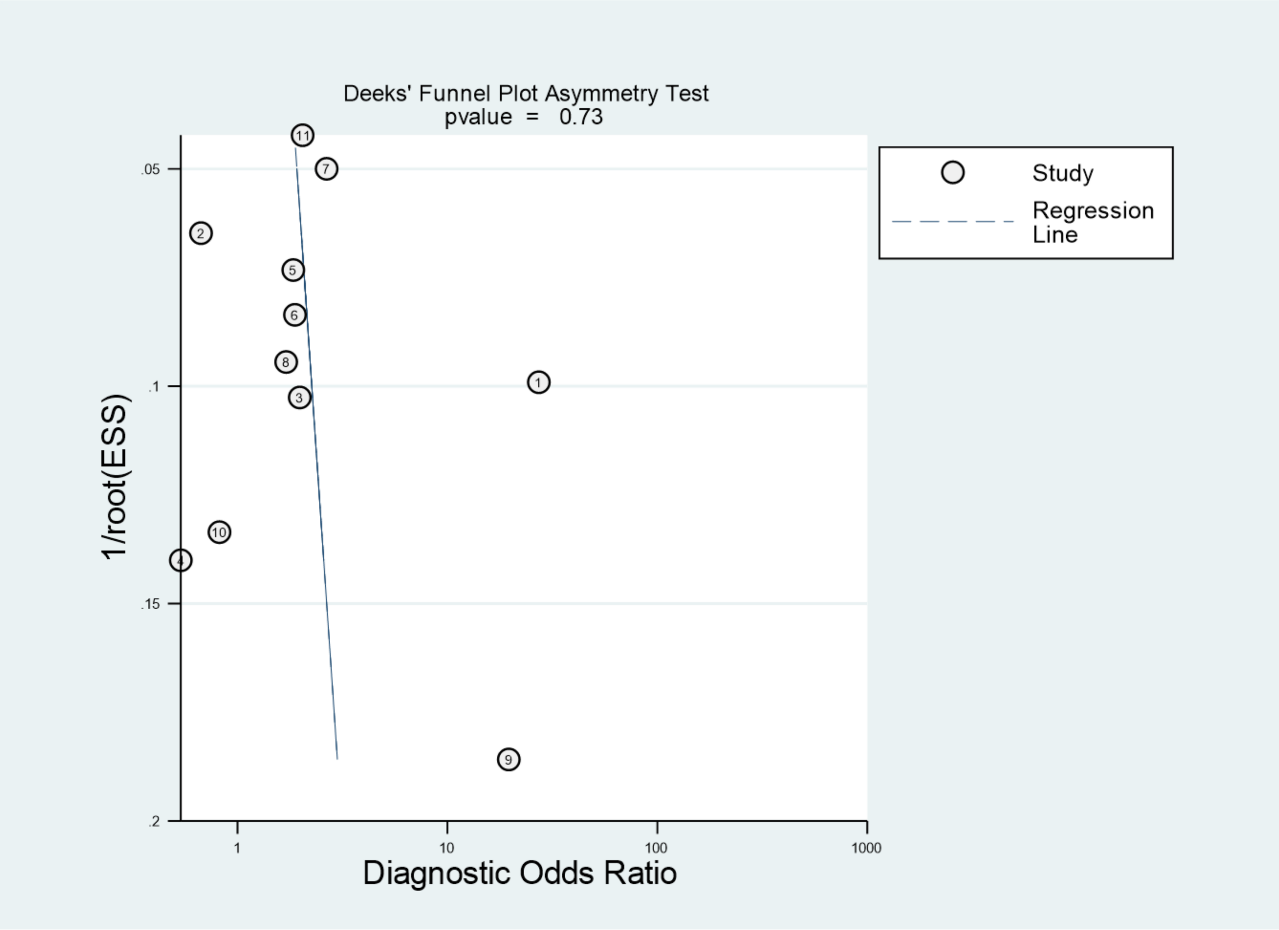
Deeks’ funnel plot identified potential publication bias of the eligible studies. ESS, effective sample size.

Deeks’ tests revealed no significant publication bias among the included evaluation pooled results of the overall performance of Formosa score (*p* = 0.73), as shown in Figure 5. *I*^2^ results revealed significant between-study heterogeneity in the pooled sensitivities (*I*^2 ^= 85.04%) and specificities of the Formosa score (*I*^2 ^= 97.65%).

### 3.6. Quality of included studies

**Figure 6:**
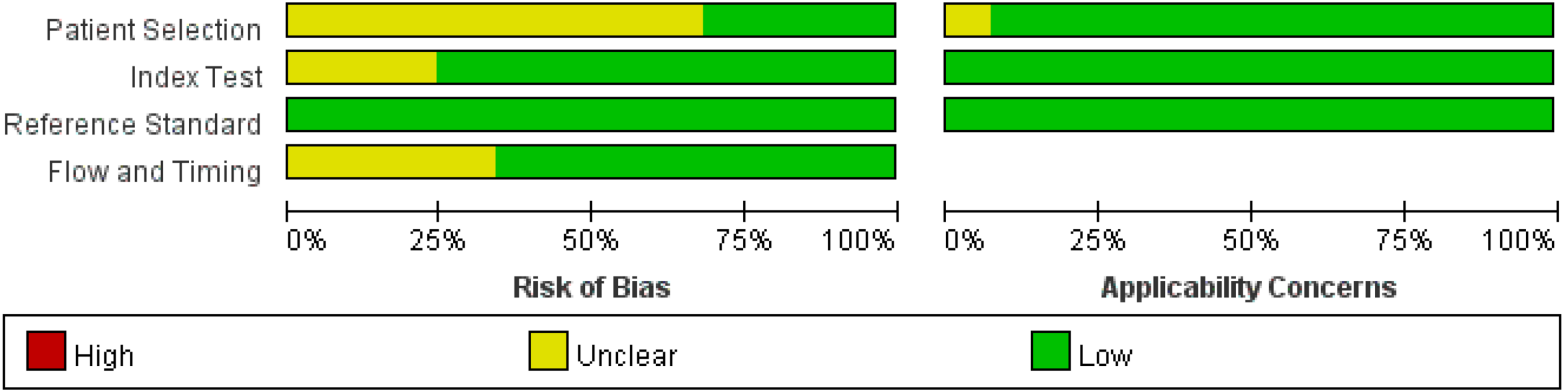
Bar graph for overall risk of bias and clinical applicability evaluated by the Quality Assessment of Diagnostic Accuracy Studies (QUADAS-2) tool.

Nine studies (22.0%) had different numbers for each score, or the number of patients enrolled was not the same as the number of patients used to calculate the scores, so they introduced bias and resulted in an unclear flow and timing (Table 1 and Figure 6) (7, 17, 28, 48–53). The included studies listed the reference standard, and the KD patients received the same reference standard. No high concerns regarding applicability of index tests, reference standard, or patient selection were observed. Only a few studies adopted prospective designs (30, 33, 44). Therefore, enrolling a consecutive or random sample of patients was hard for retrospective studies. One study used a case–control design and produced an abnormally high IVIG-resistant rate (54). Regarding the Formosa score, researchers identified IVIG-resistant patients in the clinical data including physical examination (lymphadenopathy) and laboratory, while other scores did not adopt the use of the physical examination, which may influence the diagnostic accuracy of the index test (17).

## 4. Discussion

To the best of our knowledge, this study is the first diagnostic meta-analysis to focus on comparing different scores for predicting IVIG resistance in KD patients. The identification of patients at high risk for IVIG resistance at the time of presentation is of significant benefit and may allow clinicians to identify those who may benefit from more intensive monitoring of their condition and who may require treatment modulation during the acute phase, with the potential addition of other anti-inflammatory agents to the conventional IVIG treatment to protect them from ongoing CAL. Many scoring systems have been used to predict the risk of IVIG resistance. However, the prediction efficacies of these scoring systems vary considerably. The results of five head-to-head studies suggested significant variations without consistent conclusion (30, 34, 40, 44, 46). Creating new scores for IVIG-resistant prediction is becoming an increasingly popular field of study. Since obtaining head-to-head evidence is difficult, diagnostic network meta-analyses are useful for incorporating direct and indirect comparisons with these scores (20).

In this diagnostic meta-analysis study, we evaluated the prediction efficacies of IVIG resistance through four existing scores, such as Egami, Formosa, Kobayashi, and Sano, based on their reported sensitivity and specificity in relation to clinical parameters of the risk of IVIG-resistant KD. This meta-analysis of 41 articles including 21,389 patients with KD showed that the Formosa score demonstrated the highest sensitivity in predicting IVIG resistance. The pooled sensitivity and specificity for the most commonly reported predicting tools (Egami, Formosa, Kawasaki, and Sano scores) ranged from 0.36 to 0.76 and from 0.46 to 0.83, respectively.

This bivariate network analysis faced some limitations. The meta-analysis estimated high heterogeneity for the Formosa score. The results of the Formosa score vary widely among different studies, with particularly high sensitivity in a Taiwanese study (AUC 0.84) and very low sensitivity in a Turkish study (Tables 1, 2) (12, 17, 34, 45, 46). Such a discrepancy implies that each region needs its own score, especially where the prevalence is high. The Formosa score has the potential to modify clinical practice and improve health outcomes if identifying a specific population improves the AUC. As the research on the Formosa score was conducted in China and Turkey, we could not apply our findings to other regions. The false-positive rate of the Formosa score was relatively high, so unnecessary medical intervention due to low specificity might occur. The Formosa score helped reduce unnecessary medications for patients responsive to IVIG when the score was negative.

Patients at high risk of IVIG resistance may receive adjunctive treatment to reduce coronary lesions and thus also cardiovascular morbidity. In the process of developing drugs, a good prediction score is necessary, and the Formosa score provides a good option of sensitivity to enroll more participants in trials since only 10%–20% of KD patients have IVIG resistance. More research is needed to analyze which group has a higher sensitivity and specificity of the Formosa score. Since the verification of many ethnic groups found that the Asian scoring systems were not applicable, many more accurate scoring systems have been developed (31). However, these prediction models showed unsatisfactory results when applied to Chinese, French, Iranian, Portuguese, Thai, and other populations (49, 51, 53, 55). Future network meta-analyses must also incorporate the accuracy of the new scores after new scores have undergone a certain degree of validation around the world.

## 5. Conclusion

Patients at high risk of IVIG resistance may receive adjunctive treatment to reduce coronary lesions and thus also cardiovascular morbidity. Among all of the included studies, we found that the Formosa score had the best sensitivity (0.76) but unsatisfactory specificity (0.46) for predicting IVIG resistance in Kawasaki disease.

## Data Availability

The original contributions presented in the study are included in the article, and further inquiries can be directed to the corresponding author.
